# Cyberchondria During the Coronavirus Pandemic: The Effects of Neuroticism and Optimism

**DOI:** 10.3389/fpsyg.2020.567345

**Published:** 2020-10-30

**Authors:** Alexandra Maftei, Andrei Corneliu Holman

**Affiliations:** Department of Psychology, Alexandru Ioan Cuza University, Iaşi, Romania

**Keywords:** cyberchondria, coronavirus, neuroticism, optimism, age

## Abstract

Health anxiety during the current coronavirus pandemic can be a serious psychological issue, amplified by the medical uncertainty around this disease and social isolation. As older people are especially at risk of becoming severely ill, it is important to examine the personal factors that make members of this age group more prone to health anxiety. Previous studies indicated that cyberchondria, i.e., the repeated online search for medical information, exacerbates health anxiety. The present research investigated the effect of two opposing traits, optimism and neuroticism, on cyberchondria during the COVID-19 pandemic. The associations of cyberchondria with demographic factors (age, gender, and education) were also examined. A sample of 880 participants, aged 15–67, 65% of whom were female, participated in an online survey. Results show that neuroticism, age, and being female are positively associated with cyberchondria. Optimism was found to be related to cyberchondria, but this effect was qualified by a significant interaction with age. Further analysis revealed that the effect of optimism was significant only in the highest age group. Moreover, among these elderly participants, the psychologically protective influence of optimism against cyberchondria emerged as larger than the opposite effect of neuroticism. This demonstrates the mental benefits of encouraging a positive outlook on the current health crisis and on one’s personal resilience in facing it, especially among the elderly. Conversely, among people who use the Internet as a major source of medical information, those high in neuroticism may be more prone to cyberchondria.

## Introduction

The coronavirus disease-2019 (COVID-19) emerged in December 2019, spread rapidly across the globe, and brought major changes to our lives, with countries all over the world imposing confinement measures (e.g., lockdowns and the closure of non-essential businesses) in order to avoid a rapid, uncontrolled spread of the virus and an overwhelming of medical systems. However, at the time of writing (August 2020), almost 24 million people have been infected across the globe, and more than 800,000 have already died. The COVID-19 outbreak brought an unprecedented disruption to people’s personal and social lives, with complex psychological implications (i.e., distress, anxiety, depression, financial worry, loneliness, confusion, and anger) (Brooks et al., 2020; Lin et al., 2020; Tull et al., 2020; Zhang et al., 2020).

The information rate shared via the Internet increased significantly during the pandemic, as in similar previous health crises (Sharma et al., 2017). Using social media sites or news platforms, people accessed news and various articles about the pandemic and COVID-19 related information and shared their experiences and concerns. The medical uncertainty around COVID-19, as well as the social isolation measures, raised severe psychological concerns, and online searches for information related to specific health symptoms (e.g., loss of smell or chest pain) increased during the pandemic (Walker et al., 2020). For example, people’s searches for information about COVID-19 increased in the US by 36% 1 day after the announcement of the first COVID-19 case (Bento et al., 2020). Khasawneh et al. (2020) revealed that over 80% of medical students used social media platforms and online search engines as their primary source of information on COVID-19. In Romania, searches about symptoms, health-related issues, and treatment for COVID-19 increased exponentially, with a maximum reached in March 2020^1^ (Google Trends, accessed August 23, 2020). However, research also suggested that online information about COVID-19 also contains a large amount of misleading information (Li et al., 2020), and public health agencies should aim to control the spread of misinformation concerning the virus to be able to more efficiently manage the pandemic.

In most cases, COVID-19 (the disease caused by the novel coronavirus) causes mild symptoms (such as dry cough, fever, or tiredness), though some people do not develop any symptoms. However, according to World Health Organization [WHO] (2020), 1 in 6 people may become seriously ill. The differences concerning the incidence and the severity of COVID-19 are multifaced and based on several complex factors (e.g., sociobiological characteristics or socioeconomic factors) (e.g., Hatef et al., 2020; Kopel et al., 2020); the elderly—especially those with pre-existing medical conditions (such as diabetes, cancer, lung disease, or high blood pressure)—are generally more prone to develop more severe cases of COVID-19 infections, compared to other groups (Fischer et al., 2020; Nanda et al., 2020; Niu et al., 2020).

## Cyberchondria and Associated Factors

Though accessing medical information using online sources is a common, useful, and accessible strategy for most people, in some cases, when online searching becomes excessive and repetitive, it can turn into a pathological behavior (i.e., cyberchondria). Vismara et al. (2020) provided a systematic review of cyberchondria (CYB), confirming its significant role in the increase of health anxiety, distress, and obsessive-compulsive related behaviors. Although there is still no consensus on the definition of CYB, most researchers agree that this type of behavior is often driven by distress or anxiety about one’s health (Starcevic and Berle, 2013), which subsequently increases both distress and anxiety (e.g., Belling, 2006; Recupero, 2010). More importantly, this search for online medical and health-related information is compulsive and hard to resist (Vismara et al., 2020).

Various international surveys suggested that almost 80% of Internet users used the Internet for medical appointments (Aiken et al., 2012; Fox and Duggan, 2013), while a sample of > 12,000 individuals suggested that almost half of the participants used the Google search engine for self-diagnosis (Mcdaid and Park, 2011). The constant coverage on both online and offline media of the COVID-19 pandemic might have contributed to an increase in health anxiety, particularly for people with CYB. The constant reminders of guidelines for preventions (i.e., wearing protective masks and gloves, washing hands, and avoiding social contact), along with updates related to the novel treatments for COVID-19, and infection and death rates, fueled health anxiety and exacerbated behaviors associated with CYB (Farooq et al., 2020; Hongbo et al., 2020).

Data suggests that younger individuals (aged 30–44) are the most active users when seeking health-related information via the Internet (Andreassen et al., 2007). However, the associations between age, gender, and CYB are scarce and contradictory (Vismara et al., 2020). Some studies have found no direct relationship between age and CYB (Barke et al., 2016); meanwhile, Doherty-Torstrick et al. (2016) suggested that older participants were less likely to experience an increase in anxiety due to the search for medical information, compared to younger participants.

In terms of gender, education, and their relation to CYB, Doherty-Torstrick et al. (2016) reported no significant differences between males and females, similar to Bajcar et al. (2019) and Akhtar and Fatima (2020). Zarcadoolas et al. (2002) suggested that medical information is one of the main topics researched online by the less educated. Meanwhile, Atkinson et al. (2009) reported that higher educated women (those with a Bachelor’s degree) look for more health information online, compared to men and to less educated people.

## Dispositional Optimism, Neuroticism, and Health

Dispositional optimism refers to “the generalized, relatively stable tendency to expect good outcomes across important life domains” (Scheier and Carver, 2018, p. 1082) and is considered a stable trait over time. The effects of dispositional optimism on physical health were widely examined. For example, Carver and Scheier (1981, 1998) were among the first researchers to suggest that optimistic people engage more in efforts to fight difficulties when they experience adversity and are more likely to achieve better outcomes. Previous research suggested that individuals higher in dispositional optimism generally engage in more protective health-related behaviors than people low in this specific trait (Carvajal et al., 1998; Giltay et al., 2007; Krane et al., 2018). Scheier et al. (1989) explored the associations between dispositional optimism and an objective physical health outcome (i.e., a heart attack). They found that individuals higher in optimism were significantly less likely to suffer a heart attack during a medical procedure (such as surgery).

Additionally, other researchers confirmed the links between optimism and the progression of certain diseases (e.g., atherosclerosis, Matthews et al., 2004; coronary heart disease, Tindle et al., 2009; stroke, Kim et al., 2011). People high in dispositional optimism also seem to have higher levels of cognitive functioning following traumatic brain injury (Lee et al., 2019). Dispositional optimism seems to be as important for one’s mental health as it is for their physical health. For example, Liu et al. (2016) suggested that participants’ higher levels of dispositional optimism were associated with lower levels of perceived stress and depression. Similar findings were reported by He et al. (2016), who highlighted the mediating role of optimism on the relationship between perceived social support and depression. Finally, optimism is generally negatively associated with maladaptive coping strategies (Segerstrom, 2006), and positively correlated to self-confidence, a general better adjustment in the face of adverse and traumatic life events, and healthier coping strategies compared to individuals low in dispositional optimism (Nes, 2016; Reed, 2016). During the COVID-19 pandemic, optimism was found to be significantly associated with a higher level of preventive behaviors (Jovančević and Milićević, 2020).

According to McCrae and Costa (1994), neuroticism is a temperamental, stable predisposition toward dysfunctions “that reflects the tendency to experience negative emotions, cognitions, and maladaptive behaviors” (Bajcar and Babiak, 2020, p. 1). Neuroticism was found to be a significant risk factor for CYB by Bajcar and Babiak (2020), in line with previous research that linked neuroticism and other personality traits (i.e., extraversion and conscientiousness) to health-related seeking behaviors (e.g., Lagoe and Atkin, 2015; Jacobs et al., 2017; Fergus and Spada, 2018), which are generally mediated by health anxiety (e.g., Lagoe and Atkin, 2015). Neuroticism might also harm a person’s immune system through “a “predisposition model,” wherein effects of stressors on the immune system are dependent on personality (Khosravi, 2020, p. 1.).

Concerning the COVID-19 pandemic, neuroticism was suggested as a marker of vulnerability to COVID-19 infection by Khosravi (2020), in line with Zajenkowski et al. (2020), and Abdelrahman (2020), who suggested that neuroticism may be positively associated with adopting social distancing to avoid potential COVID-19 infection. Additionally, higher neuroticism was related to more concerns and longer duration estimates related to the COVID-19 pandemic (Aschwanden et al., 2020), in line with similar findings (e.g., Weiss and Deary, 2020). Finally, Kroencke et al. (2020) suggested that individuals high in neuroticism might experience more negative effects during the COVID-19 pandemic.

## The Present Study

Although various studies suggested the significant associations between optimism, neuroticism, and health-related behaviors, and a growing number of COVID-19 studies point to the importance of psychological traits for predicting pandemic-related behavior, there is still a scarcity of research concerning the relationship between dispositional optimism, neuroticism, and cyberchondria. Therefore, this study aimed to investigate the effects of optimism and neuroticism on CYB during the COVID-19 crisis. Additionally, we examined the associations between CYB and age, gender, and education. We also investigated whether the effects of the two psychological traits assessed on cyberchondria are moderated by age, given the increased COVID-19–related risk faced by the elderly.

## Methods

### Procedure and Participants

We ran a web-based, cross-sectional survey at the beginning of April 2020, a few weeks after governments all over the world, including Romania, imposed numerous restrictions (i.e., social confinement) to prevent the spread of COVID-19. The study was approved by the Research Ethics Committee from the institution where the authors are affiliated. The survey was distributed to and by students enrolled at the university where the authors are affiliated, as a course credit requirement. The survey link was available for 10 days and posted in academic and social media groups, or by using other media channels (such as e-mails or other online communication groups). Participation was voluntary. Our final convenience sample consisted of 880 participants (57 were dropped from the study due to missing data). Their age varied from 15 to 67 (*M* = 34.36, *SD* = 10.17), most of them being females (64.8%), with a Bachelor’s degree (48.6%). Participants answered anonymously after being presented with an informed consent form describing the aim of the study and ensuring the confidentiality of their answers.

### Instruments

Participants answered the revised form of **The Life Orientation Test** (LOT; Scheier et al., 1994) which measures dispositional optimism on a 5-point Likert scale (1-strongly disagree, 5-strongly agree) using 10 items (out of which four were filler items). Cronbach’s alpha indicated satisfying reliability (0.715) of the scale.

We used the **Cyberchondria Severity Scale** (CSS; McElroy and Shevlin, 2014) to assess people’s excessive online searching for health-related issues, and the way this conduct affects their daily routine. Participants in our sample answered on a 5-point Likert scale (1-Never, 5-Always) to 33 items further divided into five different dimensions—*Compulsion*, *Distress, Excessiveness*, *Reassurance*, and *Mistrust of medical professionals*—assessing their related behavior within the past 2 weeks. High total scores indicated high levels of CYB. Cronbach’s alpha indicated high reliability (0.947) of the scale.

**The Neuroticism Scale** from the international personality item pool—IPIP (1996)—was used to measure psychological distress. The 10-item instrument was translated and adapted for the Romanian population by Iliescu et al. (2015). Cronbach’s alpha indicated good reliability (0.865) of the scale.

A demographic scale assessed participants’ age, gender, and education (i.e., high school, Bachelor’s degree, or postgraduate).

## Results

We computed the Spearman correlations between study variables, as the results of the Shapiro-Wilk tests of normality indicated that CYB, LOT, NS, and age are not normally distributed. These correlations and normality statistics are presented in Table 1, together with descriptive statistics on all variables. CYB was found to have significant but weak negative associations to optimism (i.e., LOT) and age, and to be positively related, albeit to the same weak order of magnitude, to neuroticism (i.e., NS) and gender, with female participants scoring higher than males.

**TABLE 1:** Pearson correlations between study variables, means, and standard deviations (*N* = 880).

	1	2	3	4	5	6	*M*	*SD*	Shapiro-Wilk W statistic
1. CSS	1	−0.26**	0.33**	−0.08*	0.10*	–0.05	2.29	0.73	0.97**
2. LOT		1	−0.59**	0.17**	0.006	0.12**	3.61	0.73	0.98**
3. NS			1	−0.18**	0.13**	–0.03	2.30	0.76	0.98**
4. Age				1	0.06	0.21**	34.36	10.17	0.98**
5. Gender					1	0.17**	35.2% males, 64.8% females		
6. Education						1	23.2% highschool, 48.6% Bachelor’s degree		

*p < 0.05; **p < 0.01. *CSS, Cyberchondria Severity Scale; LOT, Life Orientation Test; NS, Neuroticism Scale.*

Next, we investigated the effect of neuroticism, optimism, age, gender, and education on CYB through hierarchical multiple regression analysis. The three sociodemographic variables were entered in the first step, while neuroticism and optimism were entered in the second. In order to explore whether age moderates the effect of these two psychological dimensions on CYB, we introduced the interaction term (i.e., product) between neuroticism and age, as well as the interaction between optimism and age in the third step.

Results, summarized in Table 2, showed that in the third model age, gender, neuroticism, and optimism were significant predictors of CYB. However, while neuroticism was positive, as in the previous correlational analysis, the relationships of the other three variables to cyberchondria emerged as opposed to those indicated by simple correlations. Thus, when controlling the other dimensions, the standardized coefficients in the third step of the regression analysis indicate that male and older participants score higher on CYB than their female and younger counterparts. Moreover, optimism was indicated as having a positive association with CYB, but this main effect was qualified by a significant interaction between LOT and age, while the interaction term between neuroticism and age did not significantly account for cyberchondria. In order to explore the interaction between optimism and age, we analyzed the effect of optimism at each age level through multiple regression analyses. To this aim, we used the mean and standard deviation of the age distribution to split the sample into three groups.

**TABLE 2:** Hierarchical regression model for cyberchondria (CYB).

	Step 1	Step 2	Step 3

	β	β	β
**CSS**			
Age	−0.08*	–0.02	0.72*
Gender	0.11**	0.07*	0.07*
Education	−0.07*	–0.06	–0.06
NS		0.28**	0.50**
LOT		–0.07	0.32*
NS x Age			–0.26
LOT x Age			−0.73**
Δ*R*^2^	0.02**	0.10**	0.01*

*p < 0.01; **p < 0.001. LOT, Life Orientation Test; NS, Neuroticism Scale.

The results of the analysis regression of CYB on the three sociodemographic variables, neuroticism, and optimism in each of the three groups defined by their age level are presented in Table 3. Results indicate that neuroticism is a significant positive factor of CYB at every age level. Optimism did not emerge as a significant predictor in the first two age groups, but it negatively and significantly predicted CYB in participants with the highest age. Moreover, at this age level, the effect of optimism on CYB, as indicated by standardized regression coefficients, emerged as stronger than that of neuroticism. The association of education to CYB scores was found to be similar to that of optimism across the three age groups, as education emerged as a significant negative factor of cyberchondria only in participants in the highest age group.

**TABLE 3:** Hierarchical regression model for cyberchondria (CYB) at each age level.

	Lowest age (under 24 years) *N* = 166	Medium age (25–44 years) *N* = 572	Highest age (over 45 years) *N* = 142
**CSS**			
Age	−0.12	−0.06	0.10
Gender	0.10	0.05	0.08
Education	−0.14	−0.01	−0.21**
NS	0.34**	0.29**	0.18*
LOT	0.10	−0.08	−0.22*
*R*^2^	0.14**	0.13**	0.19**

*p < 0.01; **p < 0.001. LOT, Life Orientation Test; NS, Neuroticism Scale.

## Discussion

In a survey-based, cross-sectional study, we examined the effect of two opposite dispositional traits, i.e., optimism and neuroticism, and a series of demographic factors (gender, education, and age) on cyberchondria during the COVID-19 pandemic. We were also interested in the way age moderates the effects of the two psychological factors examined, given the fact that COVID-19 is especially dangerous for older people. Results suggested that optimism decreases the likelihood that people in the most vulnerable COVID-19 group (i.e., older individuals) will experience high levels of cyberchondria, emphasizing the importance of this psychological trait in overcoming the health-related anxiety that may be associated with CYB and amplified by the current crisis, in this specific age group. These findings extend the previously documented positive influence on optimism on protective health-related behaviors (e.g., Krane et al., 2018), suggesting that this active stance in the area of health management also protects highly optimistic individuals from excessive online searching for medical information during the current COVID-19 crisis, and from the psychological consequences of this behavior.

Given this benefit of optimism during the current pandemic, it is essential to consider ways in which it might be enhanced and/or pessimism reduced, especially among the elderly. For example, previous studies suggested that engaging in social activities, religious involvement, social support, physical activities, or practicing gratitude might enhance optimism and coping with adverse life situations (e.g., Greenglass et al., 2006; Giltay et al., 2007; Prati and Pietrantoni, 2009; Greene and McGovern, 2017; Progovac et al., 2017; Oberle et al., 2018; Wong et al., 2018). Health professionals and national authorities fighting the pandemic may benefit from the findings by focusing on the significant role of optimism for older people who use the Internet as a major source of medical information during the current health crisis.

The pattern of findings indicates that people high in neuroticism are especially prone to develop CYB manifestations irrespective of their age, in line with past studies on the relationship of this personality trait with CYB (Bajcar and Babiak, 2020), as well as with health anxiety and health-related behaviors (Lagoe and Atkin, 2015; Fergus and Spada, 2018). Results also suggest that when controlling for the effects of neuroticism, optimism, gender, and education, CYB is positively related to age, a result that contradicts previous findings (Doherty-Torstrick et al., 2016), while also highlighting the psychologically vulnerable status of the elderly during the COVID-19 crisis.

Besides other limits, such as its reliance on a convenience sample, which undermines the generalizability of the current findings, one of the limits of this study is that we did not measure health anxiety. This precludes us from concluding the degree to which the relationships that emerged in our results extend more generally in people’s health anxiety. Further research should investigate the relationships between age, CYB, and health anxiety in the context of the COVID-19 pandemic and its psychological effects. For instance, McMullan et al. (2019) suggested that age might moderate the relationship between health anxiety and CYB. Future studies could examine whether this pattern of findings is altered by increased health risks faced by the older population during the current pandemic. Furthermore, to increase the generalizability of the current findings, future studies should rely on non-convenient, larger samples. Longitudinal approaches might also express in more detail whether and how the evolution of the current pandemic affects the nature and direction of relationships in the main variables explored in the current research.

To conclude, this study found that optimism is a psychologically protective factor against CYB during the current health crisis in the most vulnerable age group, i.e., the elderly. This effect runs against the general influence of neuroticism, which amplifies the risk of CYB. The practical implications of the current findings are various and mainly important for policy makers, clinicians, and healthcare systems in general. For example, public policies should find effective ways to promote rapid and strategic ways to increase optimism as a protective measure against CYB during and post the COVID-19 pandemic. Additionally, as Khosravi (2020) suggested, given the important association between neuroticism and CYB, self-report scales might be useful for the initial screening of individuals high in neuroticism, to launch personality-tailored prevention campaigns that might reduce CYB and its negative consequences during the pandemic. (such as disseminating information about ways to fight the current crisis, especially among individuals of high neuroticism, using optimism-enhancing strategies, and advertising the negative impact of excessive and repetitive online searching for COVID-19 related information).

## Data Availability Statement

The raw data supporting the conclusions of this article will be made available by the authors, without undue reservation, to any qualified researcher.

## Ethics Statement

The studies involving human participants were reviewed and approved by the Institutional Review Board of Alexandru Ioan Cuza University in Iasi. Written informed consent to participate in this study was provided by the participants, and where necessary, the participants’ legal guardian/next of kin.

## Author Contributions

Both authors contributed equally to conceive and design the main goal of the study, analyze the data, and write the manuscript.

## Conflict of Interest

The authors declare that the research was conducted in the absence of any commercial or financial relationships that could be construed as a potential conflict of interest.

## Footnotes

1 https://trends.google.com/trends/explore?date=today%203-m&geo=RO&q=coronavirus%20simptome%20tratament
